# Effects of stand variables on stemflow and surface runoff in pine-oak forests in northern Mexico

**DOI:** 10.1371/journal.pone.0235320

**Published:** 2020-06-26

**Authors:** Francisco Cruz-Garcia, José Carlos Monárrez González, Aregai Tecle, Christian Wehenkel, Gustavo Perez-Verdin

**Affiliations:** 1 CIIDIR Dgo. Sigma 119, Instituto Politécnico Nacional, Durango, México; 2 Instituto Nacional de Investigaciones Agropecuarias y Forestales, Campo Experimental Valle del Guadiana, Durango, México; 3 School of Forestry, Northern Arizona University, Flagstaff, Arizona, United States of America; 4 ISIMA, Universidad Juárez del Estado de Durango, Durango, México; Georgia Southern University, UNITED STATES

## Abstract

The flow of water in temperate forests depends on the amount of precipitation, type of soil, topographic features, and forest cover, among other factors. Unlike the first three, forest cover can be modified by silvicultural treatments, the effects of which manifest in the quality and quantity of water, as well as in the transport of sediments and soil nutrients. The objective of this study was to analyze the effect of some stand variables on surface runoff and stemflow in pine-oak forests of northern Mexico. The stand variables included tree diameter at breast height, basal area, canopy cover, and volume. They were collected in eight 0.1-ha circular plots, measured in 2016 and re-measured in 2018. Nonlinear quantile regression was used to determine the best-fit relationships between the variables. Results indicated that surface runoff was most closely and inversely related to basal area. Stemflow was related to diameter at breast height, while showing no statistical significance. A stemflow funneling ratio did show an inverse, statistically-significant relationship with diameter at breast height. These results can help determine best forest management regimes compatible with the quantity and quality of water fluxes in this type of ecosystem.

## Introduction

Ecosystem services are defined as the benefits that nature provides to society [1]. Ecosystems such as forests, grasslands, croplands, coastal zones, and urban areas offer different services to society. These include provisioning services (food, water, wood, and fibers), regulation services (which affect climate, flooding, disease, waste, and water quantity and quality), cultural services (recreational opportunities, aesthetic, and spiritual values), and support services (soil formation, photosynthesis, and nutrient cycling) [1, 2]. Regulating services are obtained directly from ecosystems without any transformational process [1]. Water regulation is one of such services, which has great importance to society by providing adequate quality water and maintaining the water cycle [3, 4].

Water regulation in forest ecosystems involves the processes that take place after precipitation. These include interception, evapotranspiration, surface and subsurface flow, infiltration, soil erosion control, water quality, and groundwater replenishing, among others [1]. These water and soil movement-related processes are affected by various climatic and topographic factors, as well as by soil and vegetation cover types. In a forest system, a portion of the rain is intercepted by the top and other layers of the canopy. The intercepted rain evaporates and returns to the atmosphere, while the non-intercepted portion reaches the ground and deep parts of the soil [5]. Forest cover plays an important role in intercepting, capturing, and channeling rainfall. In addition, it is one factor that can be directly manipulated by resource managers using silvicultural practices that consist of different harvesting techniques of various intensities.

For the purpose of this study, we focused on the processes that regulate water movement, starting from the canopy, to the forest floor, and into the stream channels. This includes the processes of throughfall, stemflow, and surface runoff. Throughfall refers to the amount of water that passes directly through the forest canopy or drips from branches and leaves of trees [6]. It accounts for 60 to 90% of rainfall [7]. Stemflow is the fraction of the water that comes in contact with the forest canopy and runs down the trunks of trees and bushes, before being deposited on the ground [8, 9]. It is often ignored in rainfall studies because it is thought to be insignificant and expensive to measure, particularly when forests are composed by rough-barked trees [10–12]. Stemflow values represent between 1 to 4% of total rainfall, although some studies reported values up to 20% for certain forest types [7, 11, 12]. In many areas, particularly semi-arid ones, stemflow creates important islands of soil moisture and nutrients around the stem and contributes to streamflow and groundwater generation [7].

Surface runoff refers to the rainfall that flows over the surface of the soil directly into nearby channels and bodies of water [11]. It is often referred to as sheet flow, e.g., the water that resembles a braiding pattern of threads, without forming channels larger than rills and gullies [13]. In addition to vegetation and other surface obstructions, the rate of flow is dependent upon soil characteristics. There are numerous studies that describe the effect of soil infiltration capacity on surface runoff [14–17]. However, there are only a few studies that have addressed the impacts of vegetation cover on surface runoff in Mexico. Furthermore, there are only a limited number of studies in Mexico that have assessed the relationship between surface runoff and forest density, which, as we said above, can be manipulated through direct silvicultural treatments.

Silvicultural treatments affect hydrological fluxes. Intensive silvicultural treatments (e.g. stand thinning from above or clear-cutting) change forest density [18], eventually modifying throughfall and stemflow at both stand level and individual tree level [19], while increasing surface runoff [20]. The potential impact of the increased water from the surface flow may eventually affect site productivity and the provision or regulation of other ecosystem services (e.g., plant diversity, soil erosion control, carbon sequestration, etc.) [21, 22]. Varying levels of tree density affect water cycle components (namely interception, evapotranspiration, infiltration, and surface runoff), causing variations in water soil movement and groundwater reserves [21, 23]. For example, heavy rainfall occurrences, following highly intensive vegetation cover treatments (such as clear-cuts), result in increased surface runoff causing soil erosion, flooding, and water turbidity [23]. For short periods, large water and soil movements can modify the quality, quantity, and distribution of water resources [24].

This study used hydrological models to analyze throughfall, stemflow, and surface runoff in a managed pine-oak forest in northern Mexico. Hydrological models, which relate the flow of water to some stand variables, enable an evaluation of the impact of changes in forest cover on the water resources within a watershed [25]. The models can help determine the best forest management scenarios in places where regulation services are combined with provisioning ecosystem services. The objectives of this study were to evaluate the effects that forests, in terms of some forest tree and stand variables, have on throughfall, stemflow, and surface runoff in a temperate area of northern Mexico. The working hypotheses are that throughfall and stemflow are different depending on tree size and genus, and that stand density affects surface runoff.

## Materials and methods

**Fig 1:**
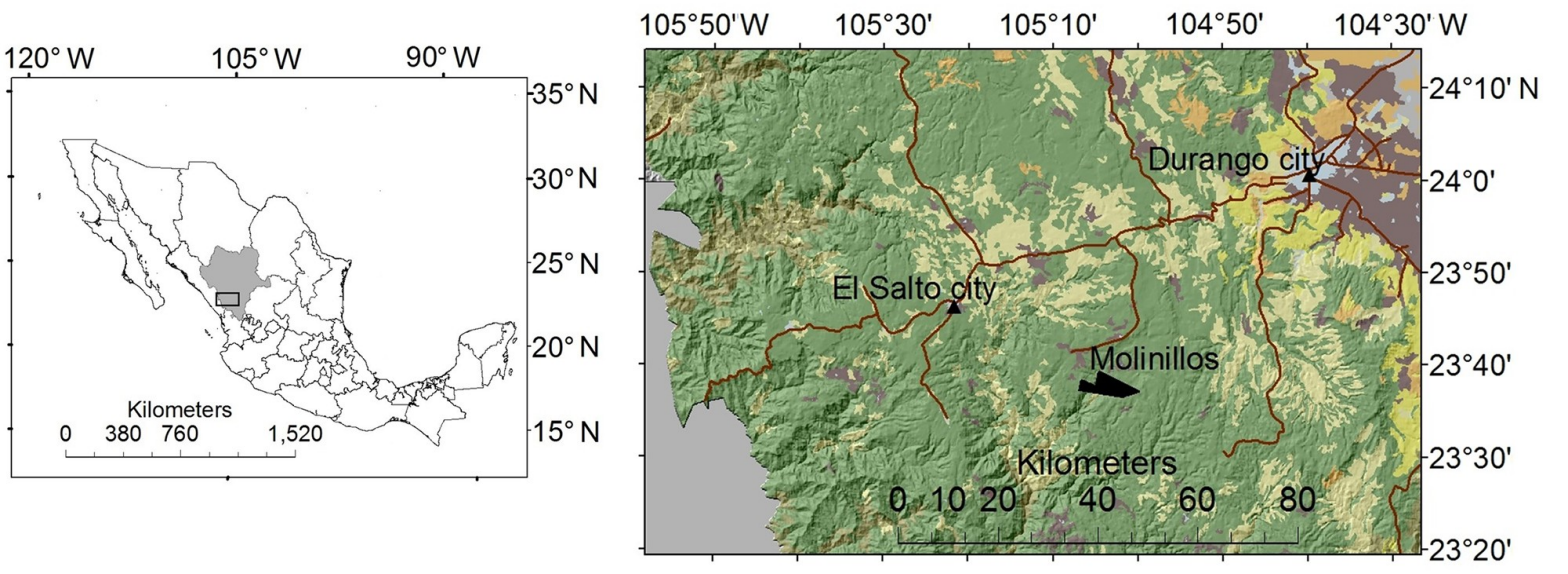
Location of the 2,866-ha study area in the state of Durango, Mexico. The ownership is called Molinillos.

The study area is located in the mountainous region of the Sierra Madre Occidental, within the municipality of Durango, which lies in the southern part of the state of Durango. The experimental site is located in a private property known as Molinillos (Fig 1). The owners of this 2,866-hectare property have played a leading role in promoting a healthy silvicultural management, biodiversity conservation, and ecotourism in the region [26]. They allowed us to conduct research and field measurements on their property. The current management plan includes the application of non-intensive tree regeneration methods (selective harvesting) as well as intensive methods (seed tree retention or clear-cuts) in different parts of the property [18, 26]. Of the total area, about 2,050 ha are under timber management, with the following treatment distribution: clear-cuts 3.5%, tree retention 14%, thinning 27%, and individual selection 55.5% [26].

The climate in the region is temperate and sub-humid, with moderate levels of rainfall in the summer and parts of December and January. In the coldest month (January), daily temperatures can reach anywhere between -3 °C and 18 °C. In the warmest month (June), daily temperatures vary between 15 °C and 35 °C. Historical records show that the mean annual temperature varies from 8 °C to 26 °C, while the annual average is 13.3 °C. The annual rainfall varies from 443 to 1450 mm, with an average of 917 mm [27].

Regional elevation ranges from 1,500 to 3,000 meters above sea level. However, the elevation in the plots is closer between 2,360 and 2,630 meters. The typical slope ranges between 20% and 60%. Runoff water flows toward the hydrographic system of the Acaponeta River basin and eventually into the Pacific Ocean. The natural, pine-oak forests include mixtures of *Pinus strobiformis*, *P*. *cooperii*, *P*. *durangensis*, *P*. *engelmannii*, *P*. *teocote*, *P*. *leiophylla*, *Quercus coccolobifolia*, *Q*. *ruogosa*, *Q*. *sideroxilla*, *Q*. *obtusata*, and *Arbutus* spp. The type of soils are Regosol, Litosol, Eutric Cambisol, and Luvisol cromico types [26].

### Tree and stand variables

The information of the tree and stand variables was obtained from eight circular plots of 1,000 m^2^. Even though there were some silvicultural treatments applied in the area, this research is not evaluating the treatment effects; instead, it is assumed that they were already applied before and their effects were observed within a range of levels of tree density.

In any case, the plots were located according to the stand density levels existing in the area. Where there were not enough sites with the desired basal areas, we waited until forest managers applied the needed prescribed silvicultural treatments. Density levels used in the study ranged from 0 to 32 m^2^ ha^-1^ of residual basal area, with a mean of 13.6 m^2^ ha^-1^. In each plot, in addition to the taxonomic identification of trees, diameter at breast height (DBH), canopy radius at each cardinal direction (*e*.*g*., N, S, W, E), and total tree height were measured for all trees with a DBH equal to or larger than 7.5 cm. Age was measured for four representative pine trees that vary on the basis of their diameter class. These measurements were used to calculate total tree volume (VOL), basal area (BA), and canopy cover (CC) at the plot level (Table 1). The goal was to determine any possible associations between throughfall (TF), stemflow (SF) or surface runoff (SR), and the stand variables. For example, the functional relationship of SR to the stand variables is described as follows:

SR=f(CC,DBH,VOL,BA) (1)

where *SR* can be replaced by SF or TF.

**Table 1:** Site and stand structural characteristics of the experimental plots.

Variable	Mean	Minimum	Maximum	Std. Dev.
DBH (cm)	17.2	7.5	85.7	11.4
Height (m)	10.0	0.3	30.0	5.5
BA (m^2^ ha^-1^)	13.6	0.0	32.4	9.5
Annual growth (m^3^ ha^-1^)	2.2	1.9	3.7	0.5
Age (years)	53.5	39.0	69.0	9.2
Canopy cover (%)	54.3	40.0	73.8	9.1
Timber volume (m^3^ ha^-1^)	147.2	101.8	188.8	28.2
Pine (%)	46.9	30.1	73.9	18.3
Oak (%)	44.7	19.3	67.2	19.1
Madrone (%)	7.72	0	36.14	14.1
Elevation (m)	2451	2366	2629	69.4
Slope (%)	41.3	10.0	66.0	13.8
Incident precipitation (mm)*	31.4	7.0	69.0	16.8
Throughfall (mm)*	19.9	1.5	69.0	16.7
Stemflow (mm)*^a^	0.37	0.001	9.86	1.07
Surface runoff (mm)*	0.26	0.003	2.54	0.38

* Average per event.

^a^ Values for all genera. Source: Present study and Perez-Verdin et al. [28].

### Throughfall

Throughfall (TH) is one of the three components of rainfall partitioning. Rainfall is the sum of throughfall, stemflow, and interception [6]. Three rain gauges with a 70-mm capacity were randomly placed in each plot under forest cover (one of them anywhere in the catchment sub-plot intended to measure surface runoff), to manually measure throughfall. Four gauges were placed in open spaces near to the plots to measure incident precipitation (Pi). The measurement period covered the months with dominant rainfalls (July to September) of 2016 and 2018. The data for 2017 were partially collected and later discarded for analysis due to logistical problems resulting from a lack of timely financial resources to continue water measurements.

### Stemflow

**Fig 2:**
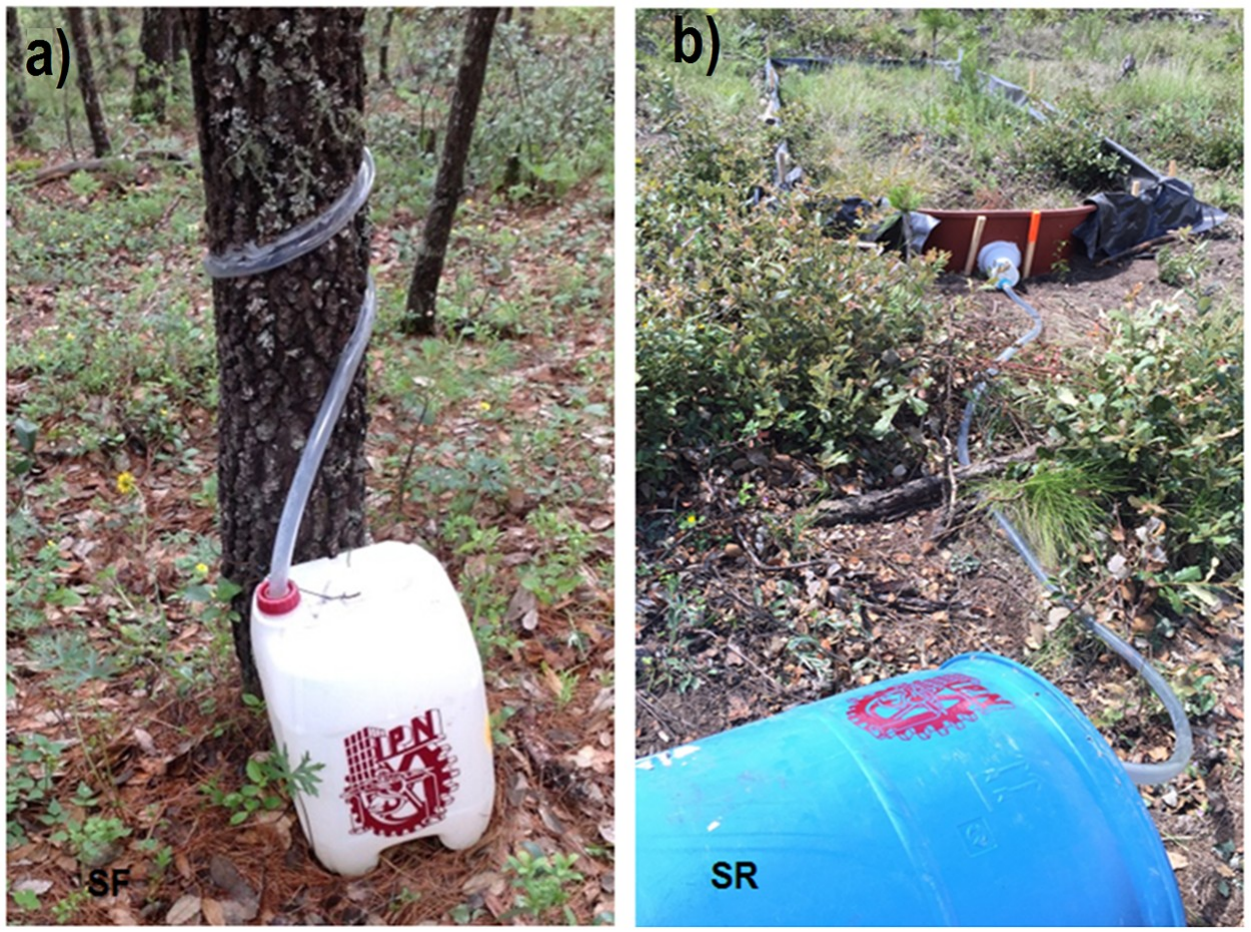
Measurement of a) stemflow (SF) and b) surface runoff (SR).

Stemflow (SF) is the other component of rainfall partitioning [29]. Along with throughfall, but excluding interception loss, they form the net precipitation that reaches the forest floor [30]. To measure and evaluate SF, four trees were selected from each 1000-m^2^ plot. The selected sample included at least one tree from each genus that consisted of pines, oaks, and madrones. If only one species was present in the plot, then all four selected trees belonged to that particular species. In total, the study used 32 sampled trees. In each tree, a rubber collar was placed around the main stem in a spiral pattern to catch and direct the water towards a 20-liter container (Fig 2) [29]. The water collected in the container was poured into a plastic beaker to measure the SF volume per tree. Measurements were recorded for every rain event or for every other event while preventing container overfill to avoid any spilling. The volume was then converted into depth (mm) using the tree crown projected area [29].

A useful measure that links precipitation, SF, and dasometric variables together in a single index is the stemflow funneling ratio (SF_r_) [6, 31]. While stemflow represents the quantity of rain water captured by a tree, SF_r_ represents the efficiency the same tree has in capturing rainfall and generating stemflow [32]. Thus, SFr measures a tree’s ability to funnel precipitation at the base, relative to its stem size and different levels of precipitation [6, 31]. The relationship is described as follows:

$${SF}_{r}=\frac{SF}{P_{i}∙B_{t}}$$ (2)

where *SF*_*r*_ is the stemflow funneling ratio (dimensionless), *SF* is the stemflow yield (L) generated by a tree (*t*), *B*_*t*_ is the basal area of the tree (m^2^), and *P*_*i*_ is incident precipitation (mm). When *SF*_*r*_ is greater than one, the funneling ratio indicates that SF is greater than the incident precipitation expected in a rain gauge occupying an area equivalent to the tree basal area [31, 32]. The funneling ratio was modeled as a function of tree variables.

### Surface runoff

Surface runoff is generated by three mechanisms, infiltration excess runoff, saturation excess runoff, and return of subsurface storm flow [14, 33]. The first mechanism (often called Hortonian runoff) occurs when the infiltration capacity of the soil is exceeded by rainfall. The second (sometimes called Dunne flow) occurs when the storage capacity of the soil is reached so that the soil cannot retain any more rain, thus resulting in surface runoff [13]. The third mechanism refers to the case when water returns from subsurface storm flow to the surface, but it is mostly detectable in larger areas or during longer evaluation periods [33]. Due to the steep slopes of the terrain, this study is concerned with the infiltration excess (Hortonian) runoff. This type of runoff resembles a shallow sheet flow formed by a braiding pattern of water threads. It can be measured by isolating a small plot on a slope corralled by metal or plastic sheets at the top, sides, and a gutter at the bottom [13]. This method has been used to evaluate the effects of forest management practices on SR [34–36].

**Fig 3:**
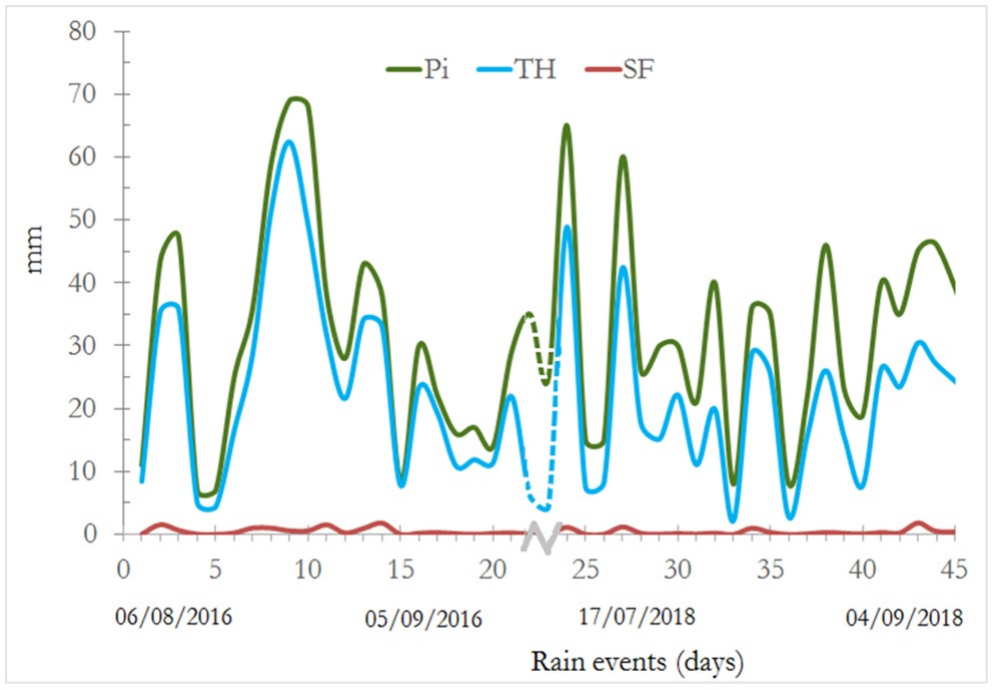
Incident precipitation (Pi), throughfall (TF), and stemflow (SF) registered in the study area (Source: Own information).

In this study, the SR experiment involved construction of 16-m^2^ sub-plots (8 x 2 m) in each of the eight 1000-m^2^ plots. The perimeter of each SR plot was surrounded by a sturdy geomembrane fence inserted into the soil to a depth of 15 cm. Another 30 cm of the fence was left above the surface to guide movement of the collected runoff into a container located on the lower part of the slope (Fig 3). Unlike metallic or wooden fences, the geomembrane can easily accommodate to the terrain irregularities. Surface runoff measurements were conducted after a precipitation event by collecting the runoff and pouring it into a plastic graduated beaker to estimate its volume (L). The standard measure for any component of the water balance is expressed in mm depth, which is obtained by dividing the water volume by the catchment area (16 m^2^). Each SR amount measured after a precipitation event was used as the main data input to find the best relationship between SR and tree density. This type of data collection approach allowed the consideration of more variable rainfall input rather than using average data.

To reduce the heterogeneity of site conditions (e.g., soil texture, grasslands, herbaceous vegetation cover, etc.) and their effects on SR, the plots were established close to each other. The average (Euclidean) distance between plots was 560 m (minimum 80 m, maximum 2.1km). Thus, stand density (as expressed in terms of basal area), would be one of the most important factors explaining the SR variability.

### Statistical analysis

Correlation estimates and scatter graphs were used to detect any preliminary associations between forest stand variables and water flow, as described in Eq 1. The Shapiro-Wilk test was used to determine the normality of the flow variables. Given that some variables were not normally distributed, parametric and non-parametric models were used to find the best fit model. The Kruskal-Wallis’ non-parametric test was also used to determine any significant differences in the SF produced by the three genera (i.e. oak, pine, and madrone).

Linear regression by quantiles models were used to find the best relationship between SR, SF, and stand variables according to Eq 1. Unlike the ordinary least squares model, quantile regression does not assume a defined distribution for the dependent variable, nor does it assume a constant variance [37]. The former model uses the conditional mean, but it does not consider the conditional variance of the response factor given a predictor [38]. In addition, quantile regression of intercept estimates are not dependent on the typical normal error distribution that ordinary least squares regression always assumes [39]. The *τ* symbol (Tau) if often used to specify the quantile levels.

Quantile regression is robust to response outliers and easily deals with rate parameter estimation for changes in the quantiles of the distribution of responses, given the independent variables [39]. This cannot be equal for all quantiles in models with heterogeneous error distributions. However, quantile regression is often criticized because it is computationally intensive and requires a fair amount of data to perform properly [38]. Nonetheless, its efficacy to model stochastic processes is recognized, particularly in those cases where data dispersion is frequent [37]. The statistical analyses were conducted using the free access software R version 3.5.1 [40], as well as the SAS^®^ system for plotting quantile regression estimates [38].

## Results

Between 40 and 50 precipitation events were registered in the main rainy seasons. For the years 2016 and 2018, the average incident precipitation was 626 mm and 704 mm, respectively. The months with the highest precipitation were August (51%), followed by September (29%) and July (20%). On average, throughfall accounted for 68% of the incident precipitation while stemflow amounts only 1.3% of the rain event (Fig 3).

### Stemflow and funneling ratio

The number of valid stemflow records for pines, oaks, and madrones were 98, 127, and 110, respectively. On average, the proportion of SF to incident precipitation was 0.6% for pines, 2.3% for oaks, and 0.9% for madrone. The Kruskal-Wallis test for the sampling period revealed significant differences among the genera (*χ*^*2*^ = 7.055, *p*<0.03). Therefore, we performed the analysis for each genus. We first attempted to analyze the relationship between SF (volume and depth) and DBH for each genus, but the results were not significant, even with transformations of variables. Interestingly, the relationships for depth-DBH and volume-DBH, were negative and positive, respectively. This contrast highlights the potential differences between volume and depth stemflow modeling. Thus, we evaluated the stemflow funneling ratio (*SF*_*r*_) as a function of DBH and obtained statistically significant results.

The median *SF*_*r*_ values for pines, oaks, and madrone were 1.27, 2.38, and 3.72 (*χ*^*2*^ = 47.71, *p*<0.01), respectively. Since these values are greater than one, they suggest that a tree funnels more water to its base than what would be expected if a rain gauge of equal cavity area had occupied the same basal area as the tree trunk [6]. In addition, for all species combined, the median *SF*_*r*_ value for large trees (i.e., DBH ≥30 cm) was 0.10, while for small trees was 1.37 (*χ*^*2*^ = 75.05, *p*<0.01). A quantile regression model was eventually adjusted for each genus. The quantile regression equation for the *SF*_*r*_ was:

$${SF}_{r}=\beta_{0}+\beta_{1}(\text{log}\;DBH)+\varepsilon$$ (3)

**Fig 4:**
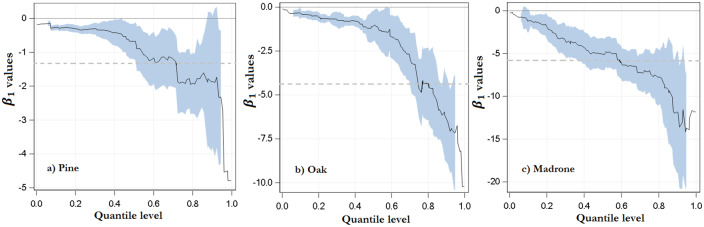
Quantile regression coefficients for the parameter DBH and 95% confidence limits as a function of quantile levels. The coefficients were adjusted to estimate the stemflow funneling ratio (Eq 3) for each genus. The dashed line in each figure represents the ordinary least squares estimate of the conditional mean effect.

Coefficient values for BA were negative for all species and quantiles (Table 2). This indicates that the stemflow funneling ratio decreases as tree diameter increases. This relationship is similar to the one depicted by SF depth (mm) and DBH, though as we said earlier, it was not significant. Table 2 shows the results of quantile regression for 0.1, 0.5, and 0.9 levels; however, as in many cases of quantile regression applications [39], we estimated the coefficients for all quantiles calculated between these numbers. Fig 4 shows the entire grid for the DBH parameters in the interval (0, 1). The shaded blue area represents the 95% confidence limits for the quantile regression estimates. For all three genera, the slope estimates (*β*_1_) decrease exponentially as the quantile level increases. Moreover, this figure reveals that, for all types of genera, the parameter estimates and confidence limits for DBH are negative across the majority of quantile levels, which suggests that, although they are always negative, they exhibit different rates of change. In the case of oaks, the slope estimates change from -0.32 in the 0.1 quantile (*p*<0.01) to -6.34 in quantile 0.9 (*p*<0.01). The same exponential relationship, but positive, occurs for the intercept estimates (*β*_0_). Both slope and intercept estimates differ across quantiles because the variance in *SF*_*r*_ changes as a function of DBH. Thus, DBH not only has an impact in the median of *SF*_*r*_, but also in its variance. This variation cannot be observed using the ordinary least squares method, since only the value for the conditional mean is obtained (dashed line, Fig 4).

**Table 2:** Quantile regression estimates and goodness-of-fit statistics for the relationship between stemflow funneling ratio and diameter at breast height (cm) in Molinillos, Mexico.

Genus	Quantile levels (*τ*)	Parameter*	Estimate	SE	Pr(>|t|)	Pseudo-R^2^
Pine (*n* = 98)	0.1	*β*_1_	-0.27	0.04	<0.01	0.66
		*Β*_0_	0.93	0.15	<0.01	
	0.5	*β*_1_	-0.68	0.25	0.03	0.36
		*Β*_0_	2.66	0.89	0.01	
	0.9	*β*_1_	-1.86	0.97	0.05	0.65
		*Β*_0_	8.67	3.55	0.02	
Oak (*n* = 127)	0.1	*β*_1_	-0.32	0.09	<0.01	0.51
		*Β*_0_	1.08	0.32	<0.01	
	0.5	*β*_1_	-1.09	0.44	0.02	0.36
		*Β*_0_	3.84	1.43	<0.01	
	0.9	*β*_1_	-6.34	1.25	<0.01	0.91
		*Β*_0_	25.16	4.16	<0.01	
Madrone (*n* = 110)	0.1	*β*_1_	-1.19	0.69	0.09	0.50
		*Β*_0_	3.58	1.79	0.05	
	0.50	*β*_1_	-5.03	0.93	<0.01	0.36
		*Β*_0_	15.8	2.63	<0.01	
	0.1	*β*_1_	-11.82	3.63	<0.01	0.91
		*Β*_0_	37.04	9.67	<0.01	

*Model parameters are based on Eq 3.

### Surface runoff

A total of 249 surface runoff (SR) records were collected during the measuring seasons (2016 and 2018), with a minimum of 0.05 l (0.003 mm) and a maximum of 40.6 l (2.54 mm). The average monthly SR was recorded in August (5.24 mm), followed by September (3.2 mm), and July (0.9 mm). Surface runoff represented about 1.9% and 0.8% of the incident precipitation for 2016 and 2018, respectively. It was mainly correlated with BA, meaning that the SR modeling was estimated with this variable alone. The quantile regression model, described by the following equation, yielded the best results:

$$SR=\beta_{0}+\beta_{1}*\text{log}\;BA+\varepsilon$$ (4)

where *SR* is expressed in mm, *BA* is the basal area per hectare (m^2^ ha^-1^), *β*_*i*_ are model parameters, and *ε* is regression error.

The relationship between SR and BA was moderately strong for the 0.5 quantile, which yielded a modest adjustment (Pseudo R^2^ = 0.45), compared to the much better results obtained from the other two quantiles (0.10 and 0.90) (Table 3). The forest vegetation in the study area is overwhelming mixed, making it difficult to separate the individual effect of each type of species on SR. Therefore, we generated an all-species model for SR. The goal was to determine the cumulative effect of managing natural, mixed forests on the amount of flow at different stand densities.

**Table 3:** Quantile regression estimates and goodness-of-fit statistics for the relationship between surface runoff (mm) and basal area (m^2^ ha^-1^) in Molinillos, Mexico (*n* = 249).

Quantile level (*τ*)	Parameter*	Estimate	SE	Pr(>|t|)	Pseudo-R^2^
0.10	*β*_1_	-0.034	0.003	<0.01	0.98
	*Β*_0_	0.115	0.009	<0.01	
0.50	*β*_1_	-0.081	0.01	<0.01	0.45
	*Β*_0_	0.320	0.05	<0.01	
0.90	*β*_1_	-0.152	0.046	<0.01	0.96
	*Β*_0_	0.818	0.138	<0.01	

*Model parameters are based on Eq 4.

**Fig 5:**
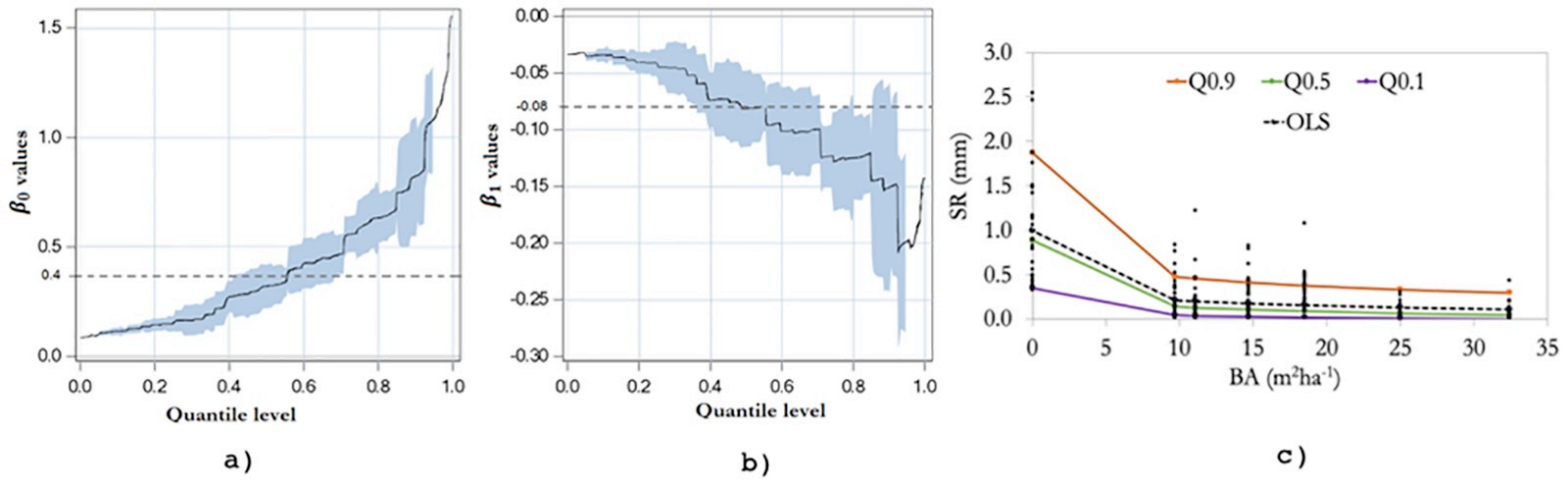
Quantile regression coefficients for the parameters used to estimate surface runoff (*n* = 249). Fig a) shows the intercept estimates and 95% confidence limits, b) shows the slope estimates and 95% confidence limits, and c) represents the regression lines for quantiles 0.9, 0.5, and 0.1. The dashed line in each figure stands for the ordinary least squares estimate of the conditional mean effect.

While positive across all quantile levels, the intercept estimates (*β*_0_) have different rates of change. They increase as the quantile levels also increase (Fig 5a). The slope estimates (*β*_1_) decrease as the proportion of quantile increases (Fig 5b). These findings suggest that there is greater data dispersion at lower values of BA and lower variability at higher values of BA (Fig 5c). Again, this is a unique advantage of quantile regression. Unlike the ordinary least squares, which coefficients are represented by horizontal, dashed lines, quantile regression allowed the estimation of parameters for the upper and lower tails of the basal area distribution.

The maximum SR values were observed in the plots in which the BA was zero, while the minimum SR values were observed in BA values higher than 20 m^2^ ha^-1^. Nevertheless, the SR remained relatively constant after BA reached 15 m^2^ ha^-1^ in all quantiles. At this level of tree density, the slope of the curve approaches zero, which implies that there is a balance with SR (Fig 5c).

## Discussion

This study was developed to analyze the influence of tree and stand variables on stemflow and surface runoff in a temperate forest area in northern Mexico. The results confirm that stemflow is affected by tree diameter and it significantly varies with genera. Likewise, surface runoff is strongly influenced by levels of stand density, expressed in terms of tree basal area (m^2^ ha^-1^). Some studies agree with these findings, while others do not. These contrasting viewpoints are discussed in some detail in the next section.

### Stemflow and funneling ratio

Results showed that SF was higher for oak trees, followed by madrone and pine species. Similar results were presented by Pérez-Suarez, et al. [41] who reported that SF in oak forests was 25% higher than in pine forests, and up to 22% higher than in mixed forests in central Mexico. Cantú-Silva and González-Rodríguez [42] found that the proportion of SF to incident precipitation was 0.6%, 0.5%, and 0.03% for pines, oak, and mixed pine-oak stands, respectively. The difference in the SF between genus (e.g. madrone, oak, and pine) may be influenced by the characteristics of each group (e.g., bark roughness, leaf area, and tree architecture). In particular, oak trees are part of the co-dominant forest structure, have a thick, rough bark [43], and can direct water to the stem base in greater quantities than pines and madrone.

The study did not find a clear relationship between SF and tree DBH. There is a positive, but not significant, relationship between SF volume and DBH. The relationship between SF depth and DBH is negative, but again without being significant. Theoretically, the size of the tree (expressed in terms of DBH, height), the shape and size of the canopy, and the bark and angle of insertion of the branches modify the canopy catchment area and thus affect the amount of SF produced [10, 44]. However, it seems that literature does not completely agree on how these factors affect stemflow. Martinez-Meza and Whitford [45] reported a direct relationship between SF and the canopy area in a Chihuahuan desert species. Chen et al. [46] also reported a direct relationship between SF and DBH, mostly when rainfall was less than 15 mm per hour. Above this threshold, the effect of tree size was not significant. Pérez-Suarez et al., [41] found a positive relationship between DBH and SF for oak species. Other studies like that of Marín et al. [5] did not detect a clear relationship between SF and BA. Yet, León-Peláez et al. [47] observed an inverse relationship between DBH and SF for pine and other species. Navar et al. [48] likewise, found a negative relationship between SF and DBH in a Tamaulipan thornscrub forest. These studies suggest the need to continue carrying out more research on site characteristics with varying topographic, soil, climatic, and vegetation conditions to establish their effects on SF.

Germer et al., [49] suggest that *SF*_*r*_, unlike the typical stemflow measure, offers more reliability to compare stemflow generation in concentrated point sources of water in forests with a diversity of tree sizes and species. This is because *SF*_*r*_ is normalized for basal area and precipitation. We found significant statistical results between the stemflow funneling ratio and DBH in the quantile regression analysis. Our results showed that there is an inverse, significant relationship between the tree funneling ratio and DBH, for all species and for all quantiles. Findings suggest that the *SF*_*r*_ decreases as a tree becomes larger. Many reasons can explain this inverse relationship. On one hand, as a tree grows, its bark gets thicker [50, 51]. According to Herwitz [31], bark has a greater water-holding capacity than foliar surfaces. Thick-barked trees have more interception storage capacity than thin-barked trees [11, 12]. On the other hand, larger trees transfer more precipitation to throughfall than smaller trees [11, 46]. In large trees, the probability of water reaching the soil by directly dripping from branches and leaves increases. Consequently, less water is transferred to stemflow and more to throughfall [10, 32].

Other studies also agreed that *SF*_*r*_ values are greater in small trees [32, 46, 48, 49]. The fact that small trees are more efficient in capturing water at their base, and thereby creating important islands of soil moisture, brings additional insight to future stemflow studies [49], which should include these type of trees. The presence of small trees, along with a diversity of species, may affect subsurface flow, saturation overland flow, and groundwater recharge [32, 48, 49].

A greater dispersion of *SF*_*r*_ values was also observed in small trees. For instance, the statistical range of *SF*_*r*_ for small pines (i.e. with a DBH less than 15 cm) was 10 whereas the range for large trees (DBH larger than 40 cm) was only 2. Many factors can explain this variability, among others, bark thickness, angle of branches, leaf index, and length of crown [32]. Quantile regression was able to deal with this DBH dispersion because it not only estimated the impact of DBH in the median of *SF*_*r*_, but also in its extreme values.

### Surface runoff

Overall, the SR quantile regression models were highly significant for each quantile tested (Table 3). Surface runoff is influenced by precipitation, the more of it, the more amount of water converts to SR. High intensity, short duration rainfall events produce more SR and soil loss in many vegetation types [35]. Quantile regression can eventually help predict the impact of varying levels of precipitation, including extreme rain events that occur during a period of study. Pérez-Verdín et al. [28] generated a non-linear model of SR using BA as the independent variable. They also found a negative relationship between SR and BA. However, their model was only fitted to the mean observed values, unlike our study, in which we also modeled the upper and lower quantiles.

Results indicated that the higher the tree BA, the lower the SR. Bosch and Hewlett [52] mentioned that SR increases significantly with heavy thinning and clear-cuts. Lack of vegetation cover, after intensive harvesting, reduces canopy interception and evapotranspiration resulting in larger amounts of runoff and downstream channel flows [53]. However, SR and streamflow responses to forest removals declined over time as forest and understory vegetation grew. Establishing plantations or increasing cover in areas with scarce vegetation decreases SR [54, 55]. The reason for this inverse relationship is because of the high tree density, which stimulates interception (and evapotranspiration) and decreases the amount of water reaching the forest floor. Depending on the slope and type of soil, it can further infiltrate deeper into the ground [34]. Conversely, excessive harvests reduce forest density, which in turn disturbs and exposes surface soil to rainfall, thus increasing erosion due to SR [20, 21, 36]. The consideration of other predicting variables such as soil texture, grasslands, and shrublands in future studies will help broaden the scope of SR models. Chen et al., [35] showed that some grass and shrub species can decrease SR up to 50% compared to forestlands with poor ground cover. The inclusion of this type of variables will also help analyze the effects of diverse forest structures not only on SR but on soil erosion as well.

Surface runoff remained relatively constant after BA reached 15 m^2^ ha^-1^ in all quantiles. This may suggest that there is some compatibility with timber production. Below this range, not only will SR be impacted, but so will many other ecosystem services [28]. Perez-Verdin et al., using multicriteria decision-making techniques, found that the most appropriate BA for the management of some ecosystem services, including SR, was between 17 and 21 m^2^ ha^-1^ [28], which is within the range we considered as compatible. This information can be useful to forest managers for prescribing better silvicultural treatments in this type of ecosystem.

The Hortonian SR starts when the intensity of rain begins to exceed the infiltration capacity of the soil. In our study, the lowest SR was recorded in the first days of July, which coincides with the beginning of the rainy season in the area. The SR increased with high rainfall intensities in late July, August, and September. In the beginning of the rainy season, there is a low SR due to a high infiltration capacity in response to gravity and metric potential that pulls the water down into dry soil [56]. But, as the rainy season progresses, the soil infiltration capacity starts to decrease and eventually reaches a constant that approaches the saturated hydraulic conductivity of the soil [16]. Eventually, swelling of the soil colloids and the closing of small cracks lower the infiltration capacity below the saturated hydraulic conductivity [56]. This process results in having more overland flow over the soil surface.

## Conclusions

This study was conducted to evaluate the effect of tree and stand variables on stemflow and surface runoff in temperate forests of the state of Durango, Mexico. We did not find a clear relationship between stemflow and stand variables. However, the stemflow funneling ratio was significant to tree diameter at breast height changes. The stemflow funneling ratio was greater in younger trees, suggesting the need to consider this type of trees in future stemflow-related studies. Stand density, expressed in terms of basal area, had significant effects on surface runoff. Denser stands intercept more precipitation and expose it to evaporative loss, allowing less water to turn into surface runoff. Less dense forests, on the other hand, permit precipitation to reach the ground with less impedance and produce more surface runoff. Surface runoff remained relatively constant after basal area reached 15 m^2^ ha^-1^ in all quantiles.

Overall, the models developed in this study show moderate statistical fit for both stemflow funneling ratio and surface runoff. Quantile regression is useful for predicting different scenarios concerning the upper and lower tails of the predictor distribution. In addition, the generated models are simple and practical to use, since DBH or BA are the only independent variables used. These variables are important measures for selecting the most suitable silvicultural treatments. Furthermore, by knowing the interactions of these variables with hydrological fluxes of interest, they can readily be used to help determining forest management regimes compatible with the quantity and quality of other ecosystem services in this type of ecosystem.

## Supporting information

S1 Data (XLSX)
